# Interleukin-6 and Outcome of Chronic Hemodialysis Patients with SARS-CoV-2 Pneumonia

**DOI:** 10.3390/medicina58111659

**Published:** 2022-11-16

**Authors:** Gabriele Donati, Lorenzo Gasperoni, Fulvia Zappulo, Anna Scrivo, Marianna Napoli, Federica Di Filippo, Maria Cappuccilli, Rita Mancini, Gaetano La Manna

**Affiliations:** 1 Nephrology, Dialysis and Renal Transplantation Unit, Azienda Ospedaliero Universitaria di Modena, 41124 Modena, Italy; 2 Surgical, Medical and Dental Department of Morphological Sciences, Section of Nephrology, University of Modena and Reggio Emilia, 41124 Modena, Italy; 3 Nephrology and Dialysis Unit, Infermi Hospital, AUSL Romagna, 47922 Rimini, Italy; 4 Nephrology, Dialysis and Renal Transplant Unit, IRCCS Azienda Ospedaliero-Universitaria di Bologna, 40138 Bologna, Italy; 5 St. Orsola Hospital, Nephrology Department, Alma Mater Studiorum, University of Bologna, 40138 Bologna, Italy; 6 LUM Metropolitan Laboratory, AUSL Bologna, 40133 Bologna, Italy

**Keywords:** chronic hemodialysis, COVID-19 mortality, interleukin-6, SARS-CoV-2 pneumonia

## Abstract

*Background and Objectives*: Chronic hemodialysis (CHD) patients are at increased risk of SARS-CoV-2 infection and the related complications and mortality of COVID-19 due to the high rate of comorbidities combined with advanced age. This observational study investigated the clinical manifestations of SARS-CoV-2 infection in CHD and the risk factors for patients′ death. *Materials and Methods*: The study included 26 CHD patients with SARS-CoV-2 pneumonia detected by positive RT-PCR on nasopharyngeal swabs and high-resolution computed tomography at hospital admission, aged 71 + 5.9 years, 14 of which (53.8%) were male, 20 (77%) under hemodiafiltration, and 6 (23%) on standard hemodialysis, with a median follow-up of 30 days. *Results*: Simple logistic regression analysis revealed that the factors associated with a higher risk of death were older age (OR: 1.133; 95%CI: 1.028–1.326, *p* = 0.0057), IL-6 levels at admission (OR: 1.014; 95%CI: 1.004–1.028, *p* = 0.0053), and C-reactive protein (OR: 1.424; 95%CI: 1.158–2.044, *p* < 0.0001). In the multiple logistic regression model, circulating IL-6 values at admission remained the only significant prognosticator of death. The ROC curve indicated the discriminatory cut-off value of 38.20 pg/mL of blood IL-6 for predicting death in chronic hemodialysis patients with SARS-CoV-2 pneumonia (sensitivity: 100%; specificity: 78%; AUC: 0.8750; *p* = 0.0027). *Conclusions*: This study identified a threshold of IL-6 levels at hospital admission for death risk in CHD patients with SARS-CoV-2 pneumonia. This might represent a valuable outcome predictor, feasibly better than other clinical, radiological, or laboratory parameters and preceding the IL-6 peak, which is unpredictable.

## 1. Introduction

Chronic hemodialysis patients (CHD) are known to be at increased risk of contracting communicable diseases, including COVID-19 (coronavirus disease 2019) [1,2,3]. While people have been sheltering at home worldwide to avoid contact with potentially infected individuals, patients who are in in-center dialysis rooms necessitate thrice-weekly access to the hospital for hours, exposing themselves to prolonged risky contact with other patients, healthcare staff, and travel personnel to reach the dialysis center. COVID-19 infection also had a triggering effect on the progression of chronic kidney disease to end-stage chronic renal failure with increased access to dialysis treatment [4]. Moreover, dialysis patients are commonly burdened by several comorbidities including cardiovascular disease, prothrombotic status, diabetes, malnutrition, vitamin D deficiency, advanced age, and immune dysfunction [5,6,7,8,9,10,11].

According to the WHO, the estimated mortality rate for COVID-19 (coronavirus disease 2019) ranges from 1.4% to 8%. However, these numbers do not mirror the situation in fragile populations, including patients with renal disease. Current evidence indicates three main culprits for dialysis patients: Firstly, a dramatically increased mortality rate in CHD ranging between 14% and 41%, which is much higher compared to non-dialysis-dependent subjects in the intensive care unit (26%) [3,12,13,14]. Secondly, the prolonged time to viral clearance, mainly due to uremia-related immune dysfunction, leads to a stringent oversight of these patients, thus a designed dialysis facility is needed until negativization [2]. Finally, CHD patients are characterized by a wider range of symptoms when SARS-CoV-2 infection takes place. The uremic state is known to be associated with impaired immune response, which could lead to reduced fever occurrence and milder symptoms compared to the general population. Since cough, dyspnea, and fatigue are often present in CHD patients, regardless of SARS-CoV-2 infection, some diagnoses might be missed in the process of screening [1]. It is also important to underline that the dialysis treatment itself represents an inflammatory trigger because of the blood–membrane interactions. Nonetheless, highly biocompatible dialyzers have recently been introduced to improve the removal of uremic toxins and cytokines, with a potential benefit for the control of the cytokine storm during SARS-CoV-2 infection [15].

Many reports describe the impact of COVID-19 on CHD patients, although with a high variability of clinical symptoms at disease onset [16]. Between the risk factors for disease severity, only age seems to maintain its predictive weight for death in CHD patients, and even the CCI does not seem to maintain its predictive effect on the mortality of dialysis patients among the different authors who have applied it to CHD with SARS-Cov-2 [14,16]. At the same time, the use of inflammatory markers to determine the severity of the disease upon admission to hospital is gaining ground in the general population affected by SARS-Cov-2 [17]. A meta-analysis carried out by Aziz et al. in 2020 suggests an IL-6 cut-off of more than 55 pg/mL for identifying patients more prone to develop a severe SARS-CoV-2 infection [18].

This observational study is aimed at describing the clinical manifestation of SARS-CoV-2 infection in CHD and identifying the risk factors for mortality, with particular attention to acute inflammation markers, as well as the potential role of adsorbing dialyzers to reduce the inflammatory burden during SARS-CoV-2 infection.

## 2. Materials and Methods

### 2.1. Study Design and Patients

**Figure 1:**
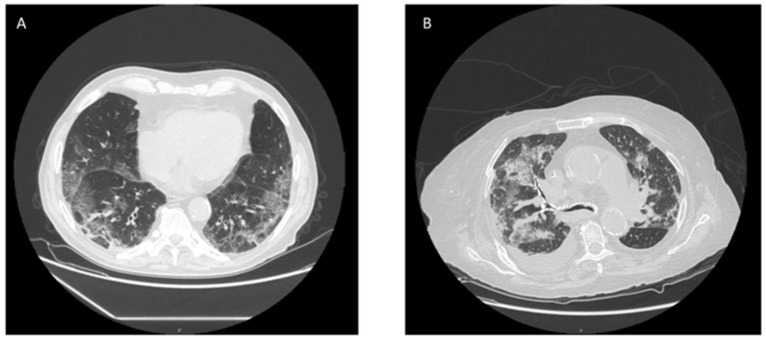
(**A**) HRCT (patient 1): Bilateral parenchymal thickening with predominantly ground-glass density. (**B**) HRCT (patient 2): Multiple areas of parenchymal lung thickening such as ground glass, bilaterally and ubiquitously distributed.

A prospective, single-center, observational study on end-stage renal disease (ESRD) patients (age ≥ 18 years) under chronic hemodialysis treatment affected by SARS-CoV-2 infection was carried out. SARS-CoV-2 infection was confirmed by a positive reverse transcriptase polymerase chain reaction (RT-PCR) on a nasopharyngeal swab and interstitial pneumonia was detected by high-resolution computed tomography (HRCT) (Figure 1). The patients were admitted to our Nephrology Dialysis and Renal Transplantation Unit from 15 March to 30 May 2020. None of the patients underwent vaccination because the anti-SARS-CoV-2 vaccination campaign started after 15 March 2021. Bicarbonate dialysis (HD) or Online hemodiafiltration (HDF) was carried out with a three-times-weekly schedule. Low-molecular-weight heparin was administered every other day after dialysis at the same dose according to the body weight of the patient (see below) to prevent thromboembolic risk. Patients with acute kidney injury, renal transplant recipients, and those who required mechanical ventilation at diagnosis were excluded. Antibiotics and low-molecular-weight heparin were administered according to the clinical needs of SARS-CoV-2 infection. The Nephrology Unit provided non-invasive ventilation (NIV) or non-invasive continuous positive airway pressure (CPAP).

At admission, the Charlson comorbidity index was recorded [19] and then body temperature, blood pressure, heart rate, peripheral hemoglobin saturation, and respiratory rate were recorded twice daily and during each dialysis.

Baseline laboratory evaluation consisted of complete blood cell count, lymphocyte count (L), creatinine kinase (CPK), lactate dehydrogenase (LDH), interleukin-6 (IL-6), complement fraction (C3 and C4), serum ferritin, C-reactive protein (CRP), and procalcitonin (PCT). IL-6 blood levels were checked every 48 h during hospitalization upon starting dialysis while arterial blood gas analysis was performed daily.

All the patients received hydroxychloroquine therapy with a dose adjusted according to ESRD (400 mg bid the first day then 200 mg/day for 5 days) and azithromycin after the first blood sampling.

Additionally, the following analytes were assayed before and after each dialysis session: C-reactive protein (CRP), PCT, C3, C4, and IL-6. Laboratory tests were carried out by commercially available tests. The Neutrophil-to-Lymphocyte ratio (NLR), Platelet-to-Lymphocyte ratio (PLR), CRP/Albumin, and IL-6/lymphocyte ratio were also assessed regarding their role in the outcome of dialysis patients [20,21].

The values measured during dialysis were corrected for hemoconcentration due to the patient′s weight loss, assuming unicompartmental behavior of the solutes described by the following formula:

Tx-corr = Tx**/**{1+ [ΔBW**/**(0.2 × BW_post_)]} (1)

where Tx is the blood solute concentration, Tx-corr is the concentration of solutes corrected for the hemoconcentration, ΔBW (body weight) is the intradialytic weight loss, and BW_post_ is the body weight at the end of dialysis [22].

The stop dialysate flow method was used to avoid access recirculation.

The primary endpoint was to evaluate circulating IL-6, alone or in combination with other biochemical and clinical parameters, as a marker able to predict patients′ mortality and clinical worsening. Clinical worsening was defined as one of the following outcomes, adapted from Goicoechea et al. [13]: (a) Death during the stay in the Nephrology ward; (b) transfer to the intensive care unit (ICU) due to severe worsening of respiratory failure requiring invasive support measures to correct hypoxemia; and (c) deteriorating respiratory failure not requiring invasive ventilatory support. Death alone was investigated as an exploratory endpoint. 

A secondary endpoint was to evaluate the removal capacity of intermittent HDF performed with the PMMA dialyzer with respect to inflammation.

The study protocol was approved by the local Ethics Committee with the code 74/2020/C/AOUBO, and each patient signed an informed written consent to participate in the study.

### 2.2. Dialysis Prescription

All the patients received three dialysis sessions per week through bicarbonate dialysis or online HDF, with the latter reserved for patients at higher risk of intradialytic hypotension, as previously suggested by Locatelli et al. [23]. The dialyzer was a high-flux PMMA filter (Filtryzer BG-U™, Toray, Tokyo, Japan) with a surface area of 2.1 m^2^, a membrane cut-off value of 20,000 daltons, and an ultrafiltration coefficient (Kuf) of 43 mL/h/mmHg. The ultrafiltration rate was established according to the clinical need. The dialysate flow was 500 mL/min. Low-molecular-weight heparin (enoxaparin sodium Inhixa™, Techdow Pharma, Milan, Italy) was used for the anticoagulation of the extracorporeal circuit at a dose of 2000 IU for patients <50 kg of body weight, 4000 IU for patients between 50 and 90 kg, and 6000 IU for patients >90 kg. Enoxaparin was administered in a single bolus upon starting dialysis in the venous bubble catcher. All dialysis sessions were carried out with the Flexya dialysis machines (Bellco/Medtronic, Mirandola, Italy).

### 2.3. Statistical Analysis

Continuous variables are presented as the mean ± standard deviation (SD) for normal distributions or as the median and interquartile range (IQR) for skewed distributions. Data comparison was performed using paired or unpaired Student′s t-tests for continuous variables with a normal distribution, Mann –Whitney and Wilcoxon tests for continuous variables with a non-normal distribution, and the Fisher test for categorical variables, as appropriate. ROC (receiver operating characteristic) curve analysis was employed to evaluate the predictive value of circulating inflammatory serum indexes with respect to the outcome. Survival analysis was used to investigate the prognostic value of basal levels of blood biomarkers. For this purpose, the population was divided according to both quartiles and median values. A *p* value below 0.05 was considered statistically significant. Calculations were performed using GraphPad Prism™ (version 8 for Windows, GraphPad Software Inc., San Diego, CA, USA).

## 3. Results

This study evaluated 26 patients aged 71 ± 5.95 years, 14 of which (54%) were male, 20 (77%) under online HDF, and 6 (23%) on standard hemodialysis. The median follow-up was 30 days (IQ range: 34.25). 

Comparisons of clinically worsening groups vs. the stable group and of survivors vs. non-survivors in terms of clinical and laboratory parameters are depicted in Table 1 and Table 2, respectively.

**Figure 2:**
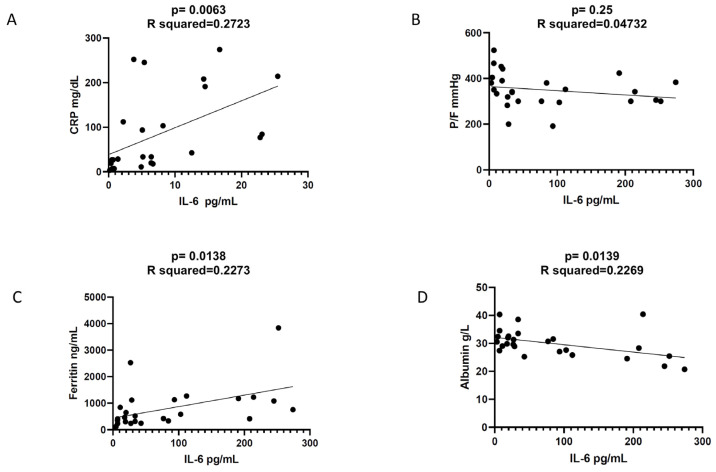
Simple linear regression analysis. (**A**) Significant correlation between IL-6 and CRP; (**B**) no correlation between IL-6 and P/F ratio; (**C**) significant correlation between IL-6 and ferritin; (**D**) inverse relationship between IL-6 and albumin.

Simple linear regression analysis showed that IL-6 levels at diagnosis correlated positively with CRP (*p* = 0.0063, R^2^ = 0.2723, correlation coefficient = 0.5218; 95% CI 0.1686 to 0.7563) and ferritin (*p* = 0.0138, R^2^ = 0.2273, correlation coefficient = 0.4767; 95% CI 0.1096 to 0.7294) and negatively with albumin (*p* = 0.0139, R^2^ = 0.2269, correlation coefficient = −0.4764; 95% CI −0.7292 to −0.1091), while no significant association was found with the PaO_2_/FIO_2_ (P/F) ratio (correlation coefficient = −0.2175; 95% CI −0.5579 to 0.1855) (Figure 2). 

### 3.1. Clinical Worsening

Among the entire population of 26 dialysis patients, 15 (58%) patients with SARS-CoV-2 pneumonia presented a clinical worsening according to the three criteria described in the method section (death, transfer to ICU, and worsening of respiratory failure without the need for invasive ventilation), while the others remained stable until discharge. In the clinical-worsening group, two patients were admitted to the ICU and two received CPAP, but they were managed in the Nephrology unit, as they were considered unsuitable for admission to the ICU due to the high rate of comorbidities. The median time interval between SARS-CoV-2 presentation and clinical worsening was 4 days (IQ range: 11 days). 

**Figure 3:**
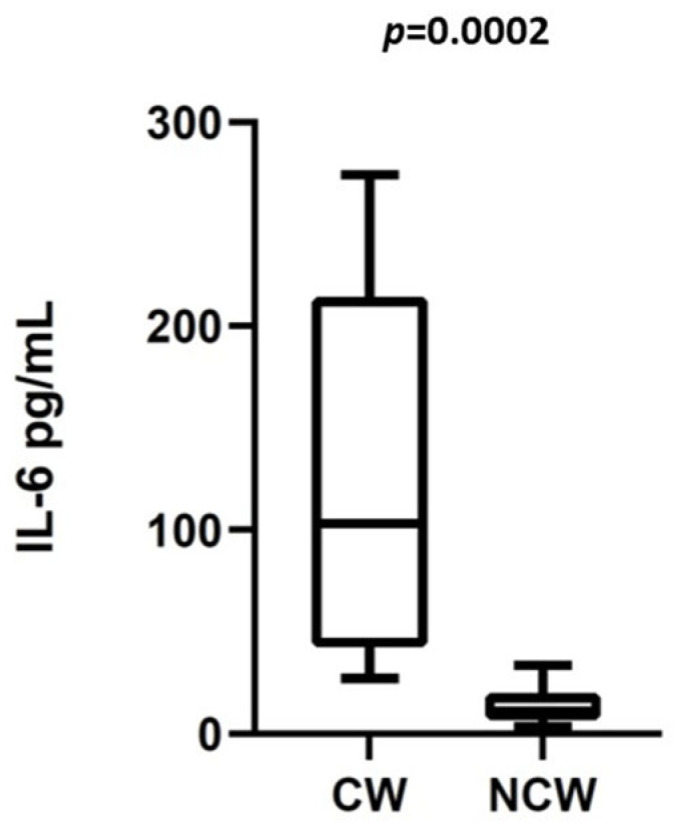
IL-6 levels at admission between 15 CHD patients in the clinical-worsening group (CW) vs. 11 CHD patients in the non-clinical-worsening group (NCW).

**Figure 4:**
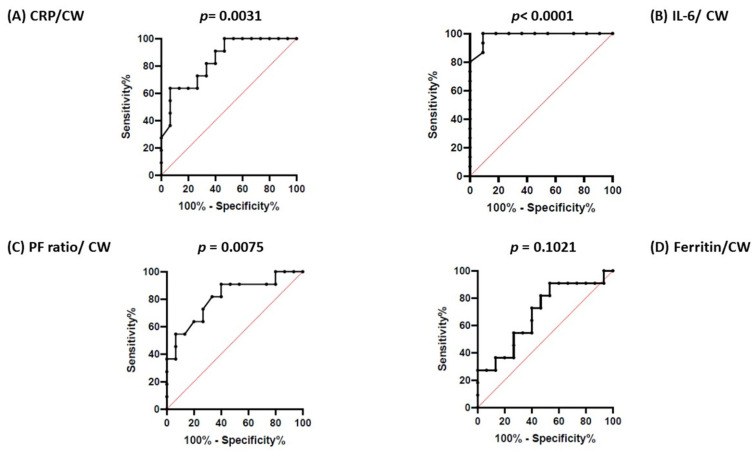
ROC curve analysis for predictors of clinical worsening (CW): CRP (**A**), IL-6 (**B**), and P/F ratio (**C**) resulted in being good predictors of CW. No relationship was found between ferritin at admission and CW (**D**).

Patients belonging to the clinical-worsening group showed, at admission, a higher rate of fever (*p* = 0.0149), a lower P/F ratio (*p* = 0.002), and higher levels of circulating IL-6 (*p* = 0.001), CRP (*p* = 0.001), IL6/L (*p* = 0.0011), and CRP/Albumin (*p* = 0.0087) (Table 1, Figure 3). Twelve out of fifteen (80%) patients in the clinical-worsening group underwent online HDF, while 10/11 (90%) in the non-clinical worsening group underwent online HDF (*p* = 0.6137). In the simple logistic regression analysis, patients with higher IL-6 levels (*p* < 0.001), a lower P/F ratio (*p* = 0.0001), higher CRP (*p* = 0.0009), and a higher IL6/L ratio (*p* < 0.0001) at baseline had a higher likelihood of belonging to the clinical-worsening group. Ferritin, age, Albumin, PCT, NLR, and PLR did not show this behavior (Table 3A). The multiple logistic regression model for the analysis of patients’ outcomes showed that neither IL-6 levels nor a low P/F ratio remained significant predictors of clinical worsening (Table 3B). In the ROC curve analysis, IL-6 at baseline resulted in being a good predictor of clinical worsening (AUC 0.9848; 95%CI: 0.9481–1.0; *p* < 0.0001). The cut-off value with the best accuracy was 27.00 pg/mL (specificity of 100% and sensibility of 90%). For the same outcome, the P/F ratio was a rather good predictor (AUC 0.8121; 95%CI: 0.6399–0.9844; *p* = 0.0075) with the best cut-off value of 326 mmHg (sensitivity of 60% and specificity of 90%). Furthermore, albumin predicted clinical worsening (AUC 0.7636; 95%CI: 0.5747–0.9526; *p* = 0.0240) while ferritin at admission was not significant (AUC 0.6909; 95%CI: 0.4804 0.9015; *p* = 0.1021) (Figure 4). In the ROC curve analysis, IL6/L at baseline resulted in being a good predictor of clinical worsening (AUC 0,9000; 95%CI 0.7705 to 1.000, *p* =0.0009): The cut-off value with the best accuracy was 27.00 (specificity of 100% and sensibility of 80%). Furthermore, CRP/Albumin at baseline resulted in being a good predictor of clinical worsening (AUC 0.8929; 95% CI 0.7647 to 1.000, *p* < 0.0013) with the best cut-off value of 0.17 (specificity of 90% and sensibility of 79%) (Figure S1, Supplementary Materials).

The days between admission and the IL-6 peak were 3.5 days (IQ range: 1–5 days) in the clinical-worsening group vs. 5 days (IQ range: 3–16 days) in the non-clinical-worsening group (*p* = 0.8970). In the clinical-worsening group, IL-6 values were 107.50 mg/dL (IQ range: 46.38–212.50 mg/dL) at admission vs. 278.00 (IQ range: 128.5–1536.00 mg/dL) when the IL-6 peak was achieved, *p* = 0.0093. In the non-clinical-worsening group, IL-6 values were 7.00 mg/dL (IQ range 4.60–19.10 mg/dL) at admission vs. 29.70 mg/dL (IQ range 7.00–73.70 mg/dL) when the IL-6 peak was achieved, *p* = 0.0313. The median time between the IL-6 peak and clinical worsening was 1 day (IQ range: 1–4 days). 

### 3.2. Patients’ Survival

This study evaluated 26 patients aged 70 ± 15 years, 14 of which (54%) were male, 20 (77%) under online HDF, and 6 (23%) on standard hemodialysis. The median follow-up was 30 days (IQ range: 34.25). 

The comparison of survivors vs. non-survivors in terms of clinical and laboratory parameters is depicted in Table 2.

**Figure 5:**
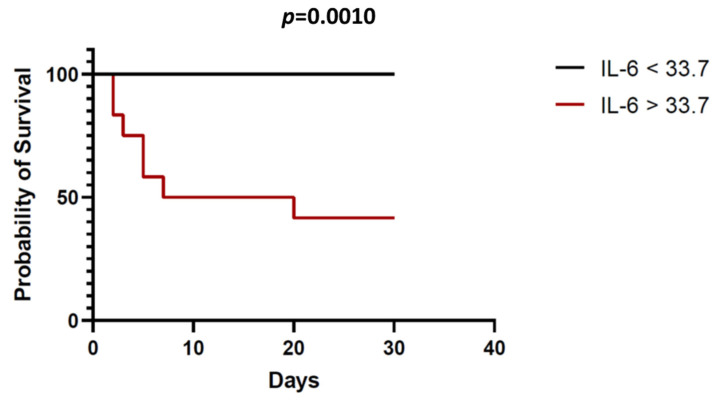
Kaplan–Meier survival analysis. Patients with IL-6 levels at admission >33.7 pg/mL had significantly lower 30-day survival in comparison to those patients with IL-6 levels < 33.7 pg/mL.

A Kaplan–Meier survival analysis was carried out after dividing the study population by the median IL-6 value at admission (33.7 pg/mL). As shown in Figure 5, the patients with IL-6 levels above the median displayed a significantly increased mortality risk at 40 days of the maximum follow-up (*p* = 0.0010).

**Figure 6:**
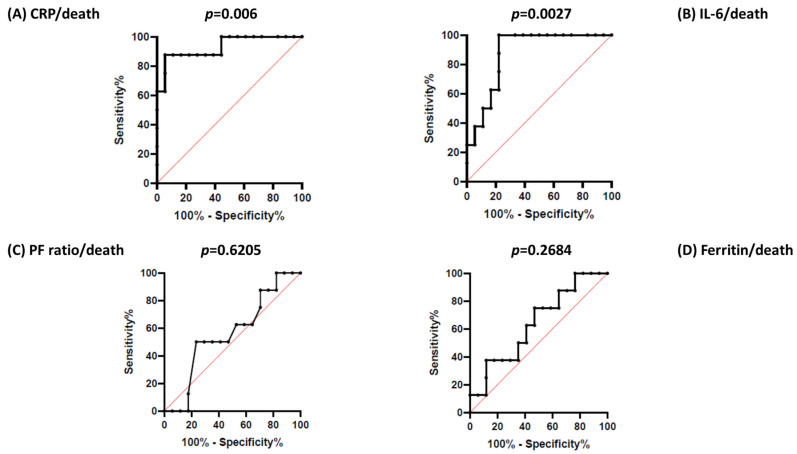
ROC curve analysis for predictors of death: CRP (**A**) and IL-6 (**B**) at admission resulted in being good predictors of death. No relationship was found between P/F ratio at admission and death (**C**), nor between ferritin and death (**D**).

Eight patients died (31%). The median time between SARS-CoV-2 presentation and patients′ death was 5 days (IQ range: 2.00–7.45 days). Patients who died displayed older age (*p*= 0.010), lower median platelet count (*p* = 0.003), higher median IL-6 levels (*p* = 0.006), and higher median CRP (*p* =0.001). The distribution of patients undergoing online HDF did not differ significantly between non-survivors and survivors [7/8 (75%) vs. 13/18 (84%); *p*= 0.6159). Simple logistic regression analysis identified the factors associated with higher death risk, namely, older age (*p*= 0.0057), higher IL-6 levels (*p* = 0.0053), and higher CRP levels (*p* < 0.0001) (Table 4A). The multiple logistic regression model regarding the outcome of death revealed that IL-6 levels remained a significant predictor of mortality independently from age (Table 4B). Receiver operating characteristic (ROC) curves used to analyze the diagnostic accuracy of IL-6 as a mortality risk predictor (AUC 0.8750; 95%CI: 0.7420–1.000; *p* = 0.0027) identified 38.20 pg/mL as the best discriminatory cut-off value (sensitivity of 100% and specificity of 78%) (Figure 2). In addition to IL-6, CRP at diagnosis showed a good predictive ability of death in CHD patients with COVID-19 (the best cut-off value of 7.4 mg/dL, sensitivity of 87%, and specificity of 94%) (AUC: 0.9306; 95%CI: 0.8175–1.000; *p* = 0.006) (Figure 6). Furthermore, IL6/L and CRP/Albumin showed a good predictive ability of death: For IL6/L (AUC 0.8897; 95%CI 0.7478 to 1.000, *p* = 0.002) in the ROC curve analysis, the best cut-off value was 95.28 (sensitivity of 94% and specificity of 75%) and for CRP/Albumin (0.8281, 95% CI 0.6614 to 0.9948, *p* = 0.01), the best cut-off was 0.30 (sensitivity of 81% and specificity of 75%) (Figure S2, Supplementary Material).

The variations in IL-6 blood levels at 72 h (calculated as the difference from baseline levels at admission) were assessed to investigate the possible relationships with survival. No difference in the median percentage of IL-6 variation was observed between the patients who survived and those who died (−0.9%, min −109, max 193 vs. −2.9%, min −50, max 2134; *p* = 0.9144). 

Nonetheless, the time interval between IL-6 levels at admission and the IL-6 peak was 3.0 days (IQ range: 1.50–3.50 days) in the non-survivors vs. 4.5 days (IQ range: 3.0–13.5 days) in the survivors (*p*= 0.2556). In the survivor group, there was a significant difference (*p* = 0.0034) between IL-6 values at baseline (27.00 mg/dL, IQ range: 6.97–98.13 mg/dL) and IL-6 peak levels (77.15 mg/dL, IQ range: 26.80–443.80 mg/dL). Conversely, in the non-survivors, the difference in IL-6 levels at baseline (147 mg/dL, IQ range: 89.05–253.30 mg/dL) and at the zenith (278.0 mg/dL, IQ range: 135.50–3375.00 mg/dL) did not reach statistical significance (*p* = 0.1250).

### 3.3. IL-6 and PCT Removal during Dialysis

**Figure 7:**
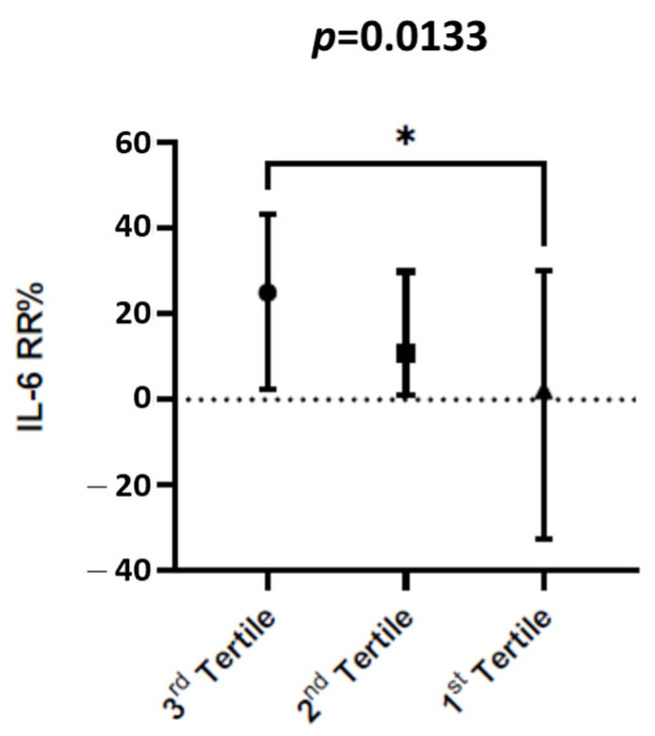
IL-6 values at admission divided into tertiles. IL-6 reduction rate is represented as mean and IQ range. In the first tertile, the median IL-6 reduction rate was 2.26% (IQ range: 62.64) during 29 HDF sessions; in the second tertile, IL-6 reduction rate was 10.71% (IQ range: 28.81) during 31 HDF sessions; in the third tertile, IL-6 reduction rate was 25.07% (IQ range: 41.05) during 28 HDF sessions. The higher the IL-6, the higher the IL-6 reduction rate (%).

IL-6 and PCT removal rates were investigated in 12/26 patients (46.1%) during 88 online HDF sessions using PMMA dialyzers. All the patients who experienced the prolonged assessment of IL-6 before and after dialysis survived. The dialysis operative results are described in Table 5. The IL-6 values were 11.40 pg/mL (IQ range of 16.95 pg/mL) upon starting dialysis vs. 10.22 pg/mL (IQ range of 11.75 pg/mL) at the end of dialysis (*p* < 0.0001). The PCT values were 0.8 pg/mL (IQ range of 1.3 pg/mL) upon starting dialysis vs. 0.46 pg/mL (IQ range of 0.74 pg/mL) at the end of dialysis (*p* < 0.0001). The removal rates of IL-6 and PCT were 13 ± 32% and 8 ± 9%, respectively. IL-6 removal rates showed no significant relationship with blood flow (*p* = 0.53) from the vascular access or convective volume of the HDF sessions (*p* = 0.20) (Figure S3, Supplementary Material). IL-6 removal rates were dependent on its baseline initial values. Pre-dialysis IL-6 levels were arbitrarily divided by tertiles: In the first tertile, the median IL-6 removal rate was 2.26% (IQ range: 62.64) during 29 HDF sessions; in the second tertile, the IL-6 removal rate was 10.71% (IQ range: 28.81) during 31 HDF sessions; and in the third tertile, IL-6 removal rates were 25.07% (IQ range: 41.05) during 29 online HDF sessions. The higher the IL-6 tertile, the higher the IL-6 removal rates (*p* = 0.0041, Figure 7).

## 4. Discussion

In this study, we evaluated uremic patients under regular chronic dialysis treatment affected by SARS-CoV-2 pneumonia, and a dramatically increased mortality rate compared to the general population (31%) was observed, as expected for CHD patients affected by SARS-CoV-2 pneumonia. Recent evidence showed that the viral load in respiratory specimens of symptomatic patients is similar to that of asymptomatic patients, thus suggesting that the viral load in respiratory specimens may not objectively reflect disease severity [24]. Likewise, the radiological signs of HRCT-proved pneumonia were comparable between patients who experienced clinical worsening and those who were clinically stable. The aim of this study was to identify risk factors upon hospital admission that can forecast clinical worsening or a patient′s death.

Regarding baseline clinical conditions, they are represented by the Charlson comorbidity index score, and we did not find significant differences between survivors and non-survivors for the Charlson score. Similarly, a study on Spanish CHD patients in two reference hemodialysis units in Madrid reported a comparable Charlson score between survivors and non-survivors [13]. 

Evidence from studies in subjects with preserved renal function suggests that SARS-CoV-2 is a severe inflammatory disease where the impact of IL-6, CRP, lactate dehydrogenase (LDH), ferritin, D-dimers, neutrophil count, and neutrophil-to-lymphocyte count on the disease progression and prognosis has been also confirmed [25].

In our CHD cohort, a significant increase in IL-6 levels and CRP was observed in patients who experienced a clinical worsening in comparison with the stable ones. These values were matched with both clinical worsening and risk of death using simple logistic regression analysis together with age, ferritin, and the P/F ratio. Then the significant variables were entered into a multivariate logistic regression analysis that confirmed IL-6 predictivity on patients′ clinical worsening and mortality: This is the first experience of the predicting ability of IL-6 on clinical worsening and death in dialysis-dependent patients. Interestingly, the IL-6 threshold for mortality at hospital admission was 38.2 pg/mL. At present, no other study reports a threshold for IL-6 circulating levels for mortality in CHD patients. 

Table S1 (Supplementary Materials) reports the IL-6 threshold for mortality and clinical worsening in the general population with SARS-CoV-2.

A large study at the Mount Sinai Institute of New York followed up with 1484 patients hospitalized for suspected or confirmed COVID-19 up to 41 days after admission: IL-6 and TNF-alpha at the time of hospitalization confirmed their predictive value on disease severity and death risk. When IL-6 was considered, its cut-off value for survival was 70 pg/mL [26]. A study by Herold et al. on 89 COVID-19 patients identified a circulating IL-6 value of 80 pg/mL as the optimal cut-off value to forecast the urgency for mechanical ventilation: When this threshold was exceeded, the median time to mechanical ventilation was 1.5 days (range: 0–4 days) [27]. Chen et al. reported that IL-6 levels detected in 48 patients with COVID-19 infection were increased almost 10-fold in comparison to COVID-free subjects. Moreover, extremely high IL-6 levels were closely correlated with the detection of viral RNAemia. Mortality appeared to be associated with an IL-6 concentration of >100 pg/mL, and this value was recognized as the threshold for a poor prognosis [28].

A rule of thumb for the nephrologist in the dialysis ward is that CHD patients are prone to chronic inflammation, which is a weighty risk factor for patients′ mortality. In a pivotal study, Bologa and colleagues showed better survival of CHD patients when their basal IL-6 levels were below 5.0 pg/mL in comparison to those with IL-6 levels of more than 11.5 pg/mL. The relative risk associated with each 1 pg/mL increase in the IL-6 concentration rises by 4.4% [29]. Therefore, the relative risk of death will increase up to 146% if we consider the difference between our IL-6 threshold in response to COVID-19 infection and Bologa′s reference IL-6 values for CHD patients (38.2–5.0 pg/mL = 33.2 × 4.4 %= 146.08%). To counteract the impaired cytokine clearance and bioincompatibility reaction to a foreign body, adsorbing more biocompatible hemodialysis membranes, such as PMMA, can be applied in CHD patients [30]. Moreover, combining adsorption and convection during HDF can be useful to control inflammation in SARS-CoV-2 CHD patients because convective transport is effective in reducing systemic inflammation through the clearance of middle-sized molecules [31]. Quiroga et al. used a high-flux PMMA dialyzer in 16 CHD patients affected by SARS-CoV-2 infection, of which 10/16 had SARS-CoV-2 pneumonia assessed by chest X-ray at admission, rather than by HRCT. The authors showed a more favorable outcome in those patients who achieve a negative IL-6 balance during the first dialysis after admission. A median IL-6 reduction rate of 25% was obtained in survivors, while that achieved in non-survivors was only 2.85% [32]. The authors used a Filtryzer^®^ NF PMMA membrane with a Kuf of 55 mL/h/mmHg, and the convective flux achieved was feasibly higher than that of our study where we used a Filtryzer BG-U PMMA dialyzer (Kuf: 43 mL/h/mmHg). Nonetheless, a sort of “anti-inflammatory” ability of PMMA was confirmed, especially in those patients with higher IL-6 levels [33]. 

## 5. Conclusions

In conclusion, the present study identified a cut-off value of IL-6 levels as an effective prognostic tool for CHD patients′ death and clinical worsening. IL-6 levels were measured at hospital admission, and they revealed the useful predictive value of patient outcomes, better than other clinical or laboratory parameters and before the achievement of the IL-6 peak, which is unpredictable. Nonetheless, the study has some weaknesses: (i) The observational design; (ii) the limited number of patients; and (iii) the lack of a control group regarding the dialysis filter used. 

Despite the mentioned limitations, our data present some strengths as well: (i) A homogeneous cohort of patients with HRCT-proven SARS-CoV-2 pneumonia; (ii) uniformity of medical and dialytic treatments; and (iii) the comprehensive clinical and laboratory analyses carried out. Larger clinical studies are needed to confirm the clinical efficacy of the IL-6 threshold in CHD patients.

## Figures and Tables

**Table 1:** Baseline characteristics of CHD patients affected by SARS-CoV-2 pneumonia.

Variables	Total (*n* = 26)	Clinical Worsening (*n* = 15)	Non-Clinical Worsening (*n* = 11)	*p* Value
**Age, years (SD)**	70 ± 15	74 ± 13.92	65 ± 17.90	0.1720
**Male, *n* (%)**	14 (53.8%)	8 (53)	6 (54)	0.9999
**Dialysis vintage, months (**IQ range**)**	26 (15.00–65.50)	44 (40–78)	21 (18–48)	0.0784
**Vascular access**				
Arteriovenous Fistula *n* (%)	13(50)	10 (55)	7 (38)	0.395
Central venous catheter n (%)	13(50)	8 (45)	4 (62)	0.740
**Primary cause of ESRD**				
Glomerulonephritis, *n* (%)	2 (8)	2 (13)	0	0.4923
Hypertensive nephropathy, *n* (%)	8 (30)	4 (26)	4 (36)	0.6828
Diabetic nephropathy, *n* (%)	2(8)	2 (6)	1 (9)	0.9999
Multiple myeloma, *n* (%)	2(8)	2 (13)	0	0.4923
Unknown, *n* (%)	8(30)	5 (33)	2 (18)	0.6576
**Comorbidity**				
Cardiovascular diseases, *n* (%)	26 (100)	15 (100)	11 (100)	NA
Diabetes, *n* (%)	14 (54)	7 (4)	5 (45)	0.9999
Cancer, *n* (%)	10 (38)	6 (40)	4 (36)	0.9999
Body mass index >30	7 (30)	7 (46)	3 (27)	0.4279
Charlson comorbidity index (SD)	7.00 (7.00–8.00)	7.00 (6.50–9.34)	6.45 (6.00–8.10)	0.5783
**Onset Symptoms**				
Fever, *n* (%)	15 (58)	12 (80)	3 (27)	0.0149
Cough, *n* (%)	3 (11)	1 (6)	2 (18)	0.558
Diarrhea or vomiting, *n* (%)	1 (4)	0	1 (9)	0.9999
Dyspnea, *n* (%)	1(4)	0	1 (9)	0.9999
Neurological worsening, *n* (%)	1(4)			
Symptomless, *n* (%)	5 (19)	2 (13)	3 (27)	0.9999
**Admission HRCT**				
Bilateral ground-glass opacity, *n* (%)	21(81)	12 (80)	9 (60)	0.176
Unilateral ground-glass opacity, *n* (%)	5 (19)	3 (20)	2 (40)	0.234
P/F mmHg, median (SD)	346 (300–380)	305 (291–367)	397 (315–433)	0.002
**Therapy**				
Hydroxychloroquine, *n* (%)	26 (100)	15 (100)	11 (100)	NA
Azithromycin, *n* (%)	24 (92)	14 (93)	10 (91)	0.9999
Steroid, *n* (%)	7(30)	6 (40)	1 (9)	0.0778
Heparin, *n* (%)	19 (73)	10 (67)	9 (82)	0.6576
**Baseline laboratory variables**				
WBC, 10^9^/L (IQ range)	7.43 (6.40–9.87)	6.66 (6.33–9.74)	8.14 (6.86–9.83)	0.4824
Neutrophils, 10^9^/L (IQ range)	5.94 (4.90–7.36)	5.60 (4.95–9.80)	6.90 (5.00–7.36)	0.123
Lymphocytes, 10^9^/L (IQ range)	0.82 (0.32–0.36)	0.80 (0.31–0.72)	0.64 (0.30–0.70)	0.159
Eosinophils, 10^9^/L (IQ range)	0.029 (0.02–0.07)	0.030 (0.02–0.07)	0.039 (0.03–0.07)	0.484
NLR (IQ range)	5,73 (4.20–9.04)	6.03 (4.12–9.32)	5.52 (4.08–9.80)	0.977
PLR (IQ range)	156.30 (62.31–285.20)	154.50 (47.08–277.00)	166.10 (104.20–304.0)	0.530
IL6/L (IQ range)	25.00 (8.00–117.00)	85.00 (29.00–312)	8.00 (4.00–17.25)	0.0004
CRP/Albumin (IQ range)	0.18 (0.02–0.50)	0.39 (0.15–0.65)	0.02 (0.01–0.15)	0.0006
Hemoglobin, g/dL (IQ range)	10.1 (9.1–11.60)	10.2 (8.50–12.00)	9.70 (9.10–10.98)	0.385
Platelet count, 10^9^/L (IQ range)	209 (138–325)	169 (118–340)	218 (192–319)	0.250
IL-6, mg/dL (IQ range)	33.7 (15.05–151.50)	103 (42.70–214.00)	11 (6.32–21.77)	0.001
Ferritin, mg/dL (IQ range)	488 (304–1119)	753 (330–1174)	404 (202–6.91)	0.141
CRP, mg/dL (IQ range)	5.12 (0.70–13.41)	8.19 (3.79–16.70)	0.72 (0.42–4.96)	0.001
**Days of hospitalization, n median (IQ range)**	33.9 (5.25–39.50)	18.5 (4.50–43-50)	32 (19.25–40.00)	0.416

The patients were also divided into patients who underwent clinical worsening and those who underwent non-clinical worsening. Continuous variables are presented as mean ± SD for normally distributed data or as median with median and IQ range (in square brackets) if non-normally distributed. Categorical variables are given as absolute numbers with percentage in brackets. ESRD, end-stage renal disease; HRCT, high-resolution computed tomography; P, partial pressure of oxygen in arterial blood; F, inspired oxygen fraction; WBC, white blood count; NLR; neutrophil-to-lymphocyte ratio; PLR; platelet-to-lymphocyte ratio; IL-6, interleukin 6; CRP, C reactive protein; IL6/L, interleukin-6-to-lymphocyte ratio; CRP/Albumin, C-reactive-protein-to-albumin ratio. The different types of variables considered have been highlighted in bold.

**Table 2:** Baseline characteristics of CHD patients affected by SARS-CoV-2 pneumonia and divided into patients who survived and non-survivors.

Variables	Total (*n* = 26)	Survivors (*n* = 18)	Non-Survivors (*n* = 8)	*p* Value
**Age, years**	70 ± 15	65 ± 16	81 ± 6	0.010
**Male, n (%)**	14 (54%)	9 (50%)	5 (62%)	0.604
**Dialysis vintage, months (IQ range)**	26 (15.00–65.5)	18 (6.00–35.50) (29.5)	60(20.00–72.70)	0.040
**Vascular access, type**				
Arteriovenous fistula, *n* (%)	13 (50.0%)	10 (55.6%)	3 (37.5%)	0.395
Central venous catheter, *n* (%)	13 (50.0%)	8 (44.4%)	5 (62.5%)	0.740
**Primary cause of ESRD**				
Glomerulonephritis, *n* (%)	2 (7.7%)	2 (11.1%)	0	n.a.
Hypertensive nephropathy, *n* (%)	8 (30.8%)	4 (22.2%)	4 (50.0%)	0.151
Diabetic nephropathy, *n* (%)	2 (7.7%)	2 (11.1%)	0	n.a.
Multiple myeloma, *n* (%)	2 (7.7%)	1 (5.6%)	1 (12.5%)	0.498
Unknown, *n* (%)	8 (30.8%)	5 (27.7%)	3 (37.5%)	0.641
**Comorbidity**				
Cardiovascular disease, *n* (%)	26 (100%)	18 (100%)	8 (100%)	n.a.
Diabetes mellitus, *n* (%)	14 (53.8%)	8 (44.4%)	6 (75.0%)	0.149
Cancer, *n* (%)	10 (38.5%)	6 (33.3%)	4 (50.0%)	0.214
Obesity (BMI > 30 kg/m^2^), *n*	7 (26.9%)	6 (33.3%)	1 (12.5%)	0.269
**Charlson comorbidity index**	7 (7.0–8.0)	8 (8.0–9.0)	7.5 (7.0–8.5)	0.112
**Onset symptoms**				
Fever, *n* (%)	15 (57.7%)	10 (55.5%)	5 (62.5%)	0.740
Cough, *n* (%)	3 (11.5%)	2 (11.1%)	1 (12.5%)	0.695
Diarrhea or vomiting, *n* (%)	1 (3.8%)	1 (5.6%)	0	n.a.
Dyspnea, *n* (%)	1 (3.8%)	2 (11.1%)	1 (12.5%)	0.918
Neurological worsening, *n* (%)	1 (3.8%)	1 (5.6%)	0	n.a.
Asymptomatic, *n* (%)	5 (19.2%)	3 (16.7%)	2 (25.0%)	0.618
**Admission HRCT**				
Bilateral ground-glass opacity, *n* (%)	21 (80.8%)	15 (83.3%)	8 (100%)	0.152
Unilateral ground-glass opacity, *n* (%)	5 (19.2%)	3 (16.7%)	0	n.a.
**Admission P/F, mmHg**	346 (310–400)	351 (350–428)	321 (300–381)	0.160
**Therapy**				
Hydroxychloroquine, *n* (%)	26 (100%)	18 (100%)	8 (100%)	n.a.
Azithromycin, *n* (%)	24 (92.3%)	17 (94.4%)	7 (87.5%)	0.524
Tocilizumab, *n* (%)	4 (15.4%)	4 (22.2%)	0	n.a.
Steroids, *n* (%)	7 (26.9%)	5 (27.7%)	2 (25.0%)	0.880
Heparin, *n* (%)	19 (73.1%)	14 (77.8%)	5 (62.5%)	0.417
**Baseline laboratory variables**				
WBC, 10^9^/L	7.43 (6.40–9.87)	7.26 (6.44–9.25)	7.59 (6.34–9.93)	0.447
Neutrophils, 10^9^/L	5.94 (4.90–7.36)	5.67 (5.00–7.11)	6.56 (4.12–7.31)	0.203
Lymphocytes, 10^9^/L	0.82 (0.32–0.86)	0.86 (0.30 -1.00)	0.64 (0.41–0.68)	0.155
Eosinophils, 10^9^/L	0.029 (0.02–0.07)	0.038 (0.030–0.042)	0.012 (0.01–0.03)	0.432
NLR (IQ range)	5.73 (4.20–9.04)	5.43 (3.78–8.80)	7.62 (4.86–10.07)	0.2907
PLR (IQ range)	156.30 (62.31–285.20)	175 (67.18–298.4)	138 (53.88–250.8)	0.4747
IL6/L (IQ range)	25.00 (8.00–117.00)	187.50 (75.25–314.5)	16.79 (3.72–42.63)	0.0011
CRP/Albumin (IQ range)	0.18 (0.02–0.50)	0.4665 (0.23–0.64)	0.05120 (0.01–0.23)	0.0087
Hemoglobin, g/dL	10.1 (9.1–11.60)	9.8 (9.0–10.77)	10.8 (8.8–13.3)	0.170
Platelet count, 10^9^/L	209 (138–325)	262 (130–278)	160 (150–210)	0.003
IL-6, mg/dL	33.7 (15.05–151.50)	23.4 (7.0–77.6)	147.3 (84.43–225.44)	0.006
Ferritin, mg/dL	488 (304–1119)	434 (300–1221)	664 (410–1188)	0.191
CRP, mg/dL	5.12 (0.70–13.41)	1.8 (0.70–5.50)	15.6 (2.0–13.4)	0.0001
**Hospital admission, *n* (%)**	25 (96%)	17 (94%)	8 (100%)	0.496
**Length of hospitalization, days**	33.9 (5.25–39.50)	44.7 (13.75–41.95)	4.75 (2.75–7.00)	0.0002

Continuous variables are presented as mean ± SD for normally distributed data or as median with IQ range (in square brackets) if non-normally distributed. Categorical variables are given as absolute numbers with percentage in brackets. BMI, body mass index; ESRD, end-stage renal disease; HRCT, high-resolution computed tomography; P, partial pressure of oxygen in arterial blood; F, inspired oxygen fraction; WBC, white blood count; NLR; neutrophil-to-lymphocyte ratio; PLR; platelet-to-lymphocyte ratio; IL-6, interleukin 6; CRP, C reactive protein; IL6/L, interleukin-6-to-lymphocyte ratio; CRP/Albumin, C-reactive-protein-to-albumin ratio; n.a., not applicable. The different types of variables considered have been highlighted in bold.

**Table 3:** Simple logistic regression analysis (A) and multiple logistic regression model (B) to predict clinical worsening in hemodialysis patients with SARS-CoV-2 pneumonia.

**A. Simple Logistic Regression Analysis**			
Variable	Odds ratio	95% CI	*p* value
IL-6	1.291	1.069–2.083	<0.001
Age	1.037	0.9865–1.101	0.1767
Ferritin	1.001	−0.0003944–0.002539	0.2303
CRP	1.369	0.09503–0.6747	0.0009
P/F ratio	0.9674	0.9333–0.9885	0.0001
PCT	2.976	1.241–15.22	0.0842
Albumin	0.8484	0.6758–1,0.10	0.0662
NLR	1.038	0.9024–1.254	0.6084
PLR	1.000	0.9958–1.005	0.9468
IL6/L	1.102	1.027–1.289	<0.0001
CRP/Albumin	58804	32.02–29465986936	0.0003
**B. Multiple logistic regression model 1**			
Variable	Odds ratio	95% CI	*p* value
IL-6	1.258	1.036 to 2.567	0.2585
P/F ratio	0.9780	0.8966 to 1.010	0.3360
**C. Multiple logistic regression model 2**			
Variable	Odds ratio	95% CI	*p* value
IL-6	1.019	1.004–1.044	0.0467
NLR	1.039	0.8924–1.288	0.6586
**D Multiple logistic regression model 3**			
Variable	Odds ratio	95% CI	*p* value
IL-6	1.356	1.073 2.608	0.1344
PLR	0.9893	0.9587–1.006	0.3199

IL-6, interleukin-6; CRP, C-reactive protein; NLR; neutrophil-to-lymphocyte ratio; PLR; platelet-to-lymphocyte ratio; CRP/Albumin, C-reactive-protein-to-albumin ratio; P, partial pressure of oxygen in arterial blood; F, inspired oxygen fraction; PCT, procalcitonin.

**Table 4:** Simple logistic regression analysis (A) and multiple logistic regression model (B) to predict mortality in hemodialysis patients with SARS-CoV-2 pneumonia.

**A. Simple Logistic Regression Analysis**			
Variable	Odds ratio	95% CI	*p* value
IL-6	1.014	1.004–1.028	0.0053
Age	1.133	1.028–1.326	0.0057
Ferritin	1.001	0.9996–1.002	0.2517
CRP	1.424	1.158–2.044	<0.0001
P/F ratio	1.001	0.9939–1.010	0.7768
PCT	1.082	0.7670–1.501	0.6120
Albumin	0.8898	0.7106–1.067	0.2160
NLR	1.016	0.8654–1.176	0.8256
PLR	0.9979	0.9911 to 1.003	0.4237
IL6/L	1.010	1.004 to 1.028	0.0018
CRP/Albumin	48.37	1.820–3619	0.0189
**B. Multiple logistic regression model**			
Variable	Odds ratio	95% CI	*p* value
IL-6	1.040	1.013–1.094	0.0326
Age	1.014	1.090–1.975	0.0413
**C. Multiple logistic regression model**			
Variable	Odds ratio	95% CI	*p* value
IL-6	1.021	1.008 to 1.041	0.0074
PLR	0.9977	0.9881 to 1.003	0.5336

IL-6, interleukin-6; CRP, C-reactive protein; P, partial pressure of oxygen in arterial blood; F, inspired oxygen fraction; NLR; neutrophil-to-lymphocyte ratio; PLR; platelet-to-lymphocyte ratio; CRP/Albumin, C-reactive-protein-to-albumin ratio; PCT, procalcitonin.

**Table 5:** Main features of 88 online HDF sessions using PMMA dialyzers in 12 patients.

Arteriovenous fistula, *n* (%)	4 (33.3%)
Central venous catheter, *n* (%)	8 (66.7%)
Time, minutes	223 ± 23
Blood flow rate, mL/min	291 ± 39
Convective volume, mL/session	16,459 ± 3411
Ultrafiltration, mL/session	2175 ± 712

Continuous variables are given as mean ± standard deviation (SD) if normally distributed or as median with median and interquartile range (IQR in square brackets) if non-normally distributed, and categorical variables are given as absolute numbers with percentages in brackets. CVC, central venous catheters; AVF, arteriovenous fistula; HDF, hemodiafiltration.

## Data Availability

The datasets used and analyzed during the current study are available from the corresponding author upon reasonable request.

## Supplementary Materials

The following are available online at https://www.mdpi.com/article/10.3390/medicina58111659/s1, Figure S1: ROC curve analysis for predictors of Clinical worsening: CRP/albumin ratio (B) and IL-6/L ratio (B) resulted good predictors of clinical worsening. No relationship was found between NLR ratio at admission and clinical worsening (C), as well as between PLR and clinical worsening (D), Figure S2: ROC curve analysis for predictors of death: CRP/albumin ratio (B) and IL-6/L ratio (B) resulted good predictors of death. No relationship was found between NLR ratio at admission and death (C), as well as between PLR and death (D), Figure S3: No correlation was found between: A) IL-6RR (%) and blood flow (Qb, mL/min) or B) IL-6RR and convective volume (L/session) during on-line HDF in 12/26 patients, Table S1: IL-6 threshold and clinical outcome in patients with SARS-CoV-2 among the general population. Sensitivity and specificity data for each of the thresholds are provided using the data of the present study. Refs. [34,35] are cited in Table S1.
