# Supplementary material for: Interleukin-6 and Outcome of Chronic Hemodialysis Patients with SARS-CoV-2 Pneumonia
Source: Medicina (Kaunas). 2022 Nov 16;58(11):1659. doi: 10.3390/medicina58111659 (PMC9699479; doi:10.3390/medicina58111659)
Supplement: Supplementary file 1 [file medicina-58-01659-s001.zip › medicina-1991919-supplementary.pdf]

"Interleukin-6 and outcome of chronic hemodialysis patients with SARS-CoV-2 pneumonia (medicina-1991919)": Supplementary materials.

Figure S1 (supplementary material). ROC curve analysis for predictors of Clinical worsening: CRP/albumin ratio (B) and IL-6/L ratio (B) resulted good predictors of clinical worsening. No relationship was found between NLR ratio at admission and clinical worsening (C), as well as between PLR and clinical worsening (D).

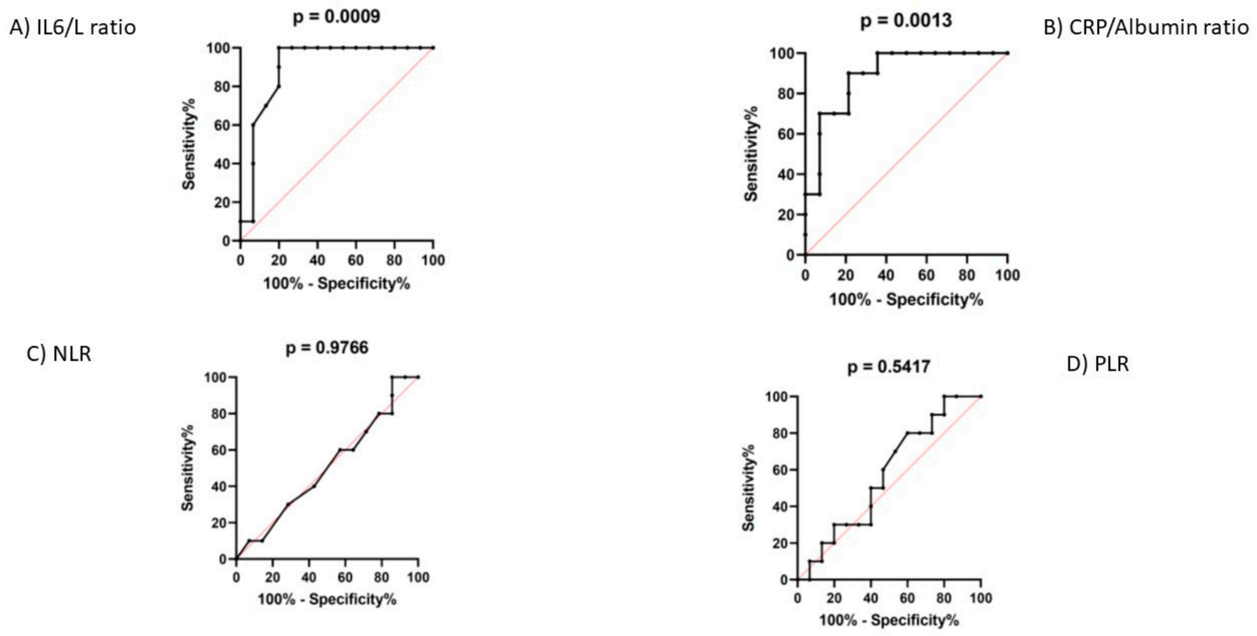

Figure S2 (supplementary material). ROC curve analysis for predictors of death: CRP/albumin ratio (B) and IL-6/L ratio (B) resulted good predictors of death. No relationship was found between NLR ratio at admission and death (C), as well as between PLR and death (D).

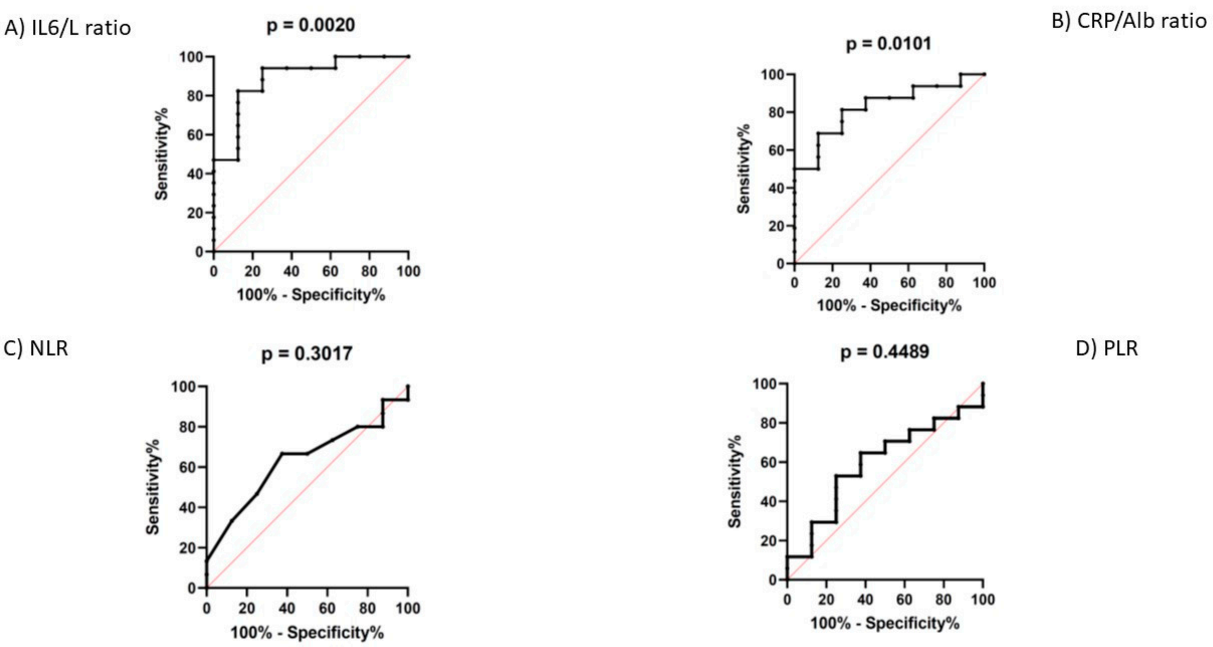

Figure S3 (supplementary material). No correlation was found between: A) IL-6RR (%) and blood flow (Qb, mL/min) or B) IL-6RR and convective volume (L/session) during on-line HDF in 12/26 patients.

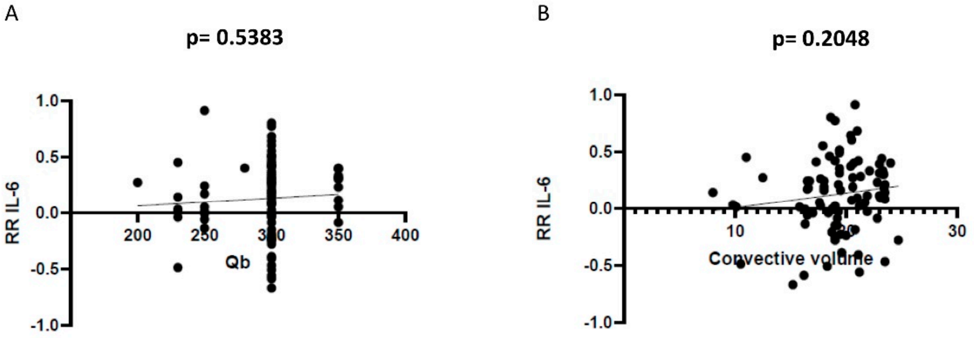

Table S1. IL-6 threshold and clinical outcome in patients with SARS-Cov-2 among the general population. Sensitivity and specificity data for each of the thresholds are provided using the data of the present study.

**Author, year**	**IL-6 threshold (pg/ml)**	**Patients (n)**	**Outcome**	**Reference**	***Sensitivity* (%)**	***Specificity* (%)**
1.Aziz et al, 2020[19]	55	1,426	Severe disease	18	87	76
2.Del Valle et al, 2020[26]	70	1,484	Mortality	26	75	76
3.Herold et al, 2020[27]	80	89	Mechanical ventilation	27	75	76
4.Chen et al, 2020[28]	100	48	Mortality	28	63	82
5.Grifoni et al, 2020[34]	25	77	Mortality	34	100	47
6.Chen et al, 2020[35]	80	273	Mortality	35	75	76
